# Injuries in Left Corticospinal Tracts, Forceps Major, and Left Superior Longitudinal Fasciculus (Temporal) as the Quality Indicators for Major Depressive Disorder

**DOI:** 10.1155/2021/2348072

**Published:** 2021-08-21

**Authors:** Ziwei Liu, Lijun Kang, Aixia Zhang, Chunxia Yang, Min Liu, Jizhi Wang, Penghong Liu, Kerang Zhang, Ning Sun

**Affiliations:** ^1^Department of Psychiatry, First Hospital of Shanxi Medical University, Taiyuan, China; ^2^School of Humanities and Social Sciences, Shanxi Medical University, Taiyuan, China; ^3^Department of Mental Health, Shanxi Medical University, Taiyuan, China

## Abstract

At present, the etiology and pathogenesis of major depressive disorder (MDD) are still not clear. Studies have found that the risk of first-degree relatives of MDD is 2–3 times that of the general population. Diffusion tensor imaging (DTI) has been previously used to explore the pathogenesis of MDD. The purpose of this study is to explore the etiology of MDD by DTI and further to explore the correlation between its clinical characteristics and the structural changes of white matter in the brain. The study included 27 first-episode, drug-naive patients with MDD, 16 first-degree relatives without MDD, and 28 healthy control subjects with no family history of MDD (HC). Results showed that the fractional anisotropy (FA) differences among the three groups were mainly in the left anterior thalamic radiation (LATR), right anterior thalamic radiation (RATR), left corticospinal tracts (LCST), forceps major (FMa), right inferior longitudinal fasciculus (RILF), and left superior longitudinal fasciculus (temporal) (LSLF(T)). Among the 6 sites, LCST, FMa, and LSLF(T) showed significant differences between MDD and First-degree relatives compared to HC. MDD patients had significant emotional symptoms, somatic symptoms, and cognitive impairment. FMa FA was significantly positively correlated with delayed memory score (*r* = 0.43, *P* = 0.031), and RILF FA was significantly negatively correlated with the FSS score (*r* = −0.42, *P* = 0.028). These results revealed that the white matter characteristics of MDD-susceptible patients were LCST, FMa, and LSLF(T) lesions, all of which may be quality indicators of MDD.

## 1. Introduction

Major depressive disorder (MDD) is characterized by cognitive impairments, functional disability, and mortality. In 2019, the prevalence of MDD in the Chinese population reached 6.8% [1, 2], and 15% of patients had suicidal behavior [3]. However, the pathogenesis of MDD is still unclear.

Genetic studies have shown that depression has familial clustering, and the prevalence of first-degree relatives is 2–3 times that of the general population. Having first-degree relatives with early/repeated episodes may increase the risk of MDD up to 6 times [4]. In the twin study, the heritability of MDD in males and females was 0.41 and 0.49, respectively, and it was found that the age of onset, number of relapses, comorbidities, anxiety, and clinical severity could predict the risk in relatives [5]. According to the high heritability, there are some diathologic changes in first-degree relatives that make them more susceptible to MDD. Meta-analysis of first-degree relatives of MDD patients showed significant differences in cognitive function. We proposed that cognitive impairment is a characteristic marker of familial aggregation of MDD [6]. It can be inferred that first-degree relatives of MDD may have similar characteristics, which may be related to the quality changes of the onset. Therefore, the task of exploring the clinical characteristics of the genetic rules of MDD is one of great significance.

Magnetic resonance imaging (MRI) is a safe and reliable neuroimaging technique. The commonly used MRI mainly includes functional magnetic resonance imaging (fMRI), structural magnetic resonance imaging (sMRI), and diffusion tensor imaging (DTI) [7]. The important principle of DTI is dispersion. The white matter of the brain has a fixed structure, which makes the dispersion of water molecules in each direction different, thereby resulting in an index called fractional anisotropy (FA). FA refers to the proportion of anisotropic components of water molecules in the whole dispersion tensor, and its value is between 0 and 1. Previous studies have shown that numerous changes in white matter fiber integrity are indicative of poor antidepressant efficacy [8]. These studies all showed abnormalities of corpus callosum (CC), capsula interna (CI), and superior longitudinal fasciculus (SLF) in MDD; however, without the ability to distinguish the quality change and the state change, the role they play is still unclear. Therefore, we hypothesized that MDD patients have white matter changes, some of which are quality indicators of MDD. We also hypothesized that the other parts are specific state changes that promote the occurrence of disease, and these white matter changes are closely related to clinical symptoms.

## 2. Materials and Methods

### 2.1. Participants

#### 2.1.1. MDD

Inclusion criteria are the following: (1) first-episode, drug-naive patients with MDD admitted to the First Hospital of Shanxi Medical University; (2) 18 ≤ age ≤ 60; (3) conformance to the Diagnostic and Statistical Manual of Disorders Fourth Edition (DSM-IV) MDD diagnostic criteria and through a Structured Clinical Interview for DSM-IV TR Axis I Disorders Patient Edition (SCID-I/P) screening [9]; (4) Hamilton Depression Scale 24 (HAMD‐24) ≥ 20; (5) no regular use of antipsychotics, antidepressants, or sedative and hypnotic drugs in the two weeks before enrollment; and (6) right-handedness. Exclusion criteria are the following: (1) a history of diseases of the nervous system, major physical diseases, or endocrine diseases; (2) a history of brain injury, coma, and other diseases that may interfere with the study; (3) other medical conditions diagnosed by the DSM-IV, including a history of alcohol or drug abuse or dependence; (4) implanted metal materials, pacemakers, etc.; (5) pregnant or lactating women; and (6) a family history of manic episodes or bipolar disorder. A total of 27 cases were enrolled.

#### 2.1.2. First-Degree Relatives

Inclusion criteria are the following: (1) biological parents, children, or siblings of above patients; (2) 18 ≤ age ≤ 60; (3) HAMD‐24 < 8; and (4) right-handedness. Exclusion criteria are the following: (1) meeting the inclusion or exclusion criteria for “MDD”; (2) severe head trauma or neonatal diseases; and (3) having a high fever convulsion in childhood or infancy. A total of 16 cases were enrolled.

#### 2.1.3. HC

Inclusion criteria are the following: (1) 18 ≤ age ≤ 60; (2) age, gender, and education level match the above two groups; (3) HAMD‐24 < 8; and (4) right-handedness. Exclusion criteria are the following: (1) meeting the inclusion or exclusion criteria for “MDD”; (2) a clear family history of mental or neurological diseases; (3) severe head trauma or neonatal diseases; and (4) having a high fever convulsion in childhood or infancy. A total of 28 cases were enrolled.

This study was approved by the Ethics Committee of the First Hospital of Shanxi Medical University.

### 2.2. Methods

#### 2.2.1. Diagnosis and Scale Evaluation

The general demographic data of the patients were collected: gender, age, education, family history, history of tobacco/alcohol use, and substance abuse. All of the scales were evaluated by the same experienced psychological evaluator. MDD should not be observed from a single perspective but must be observed from multiple perspectives of emotional experience, physical experience, and cognition [10]. We collected the following data from MDD and HC: the HAMD-24 for the patient's condition, the Snaith-Hamilton Pleasure Scale (SHAPS) for affective symptoms, the Fatigue Severity Scale (FSS) for somatic symptoms, and the Assessment of Neuropsychological Status (RBANS) for cognitive function.

#### 2.2.2. fMRI Scanning

The data were collected by Siemens 3.0 T MRI scanner and 12-channel phased array surface head coil in Shanxi Provincial People's Hospital. During the scan, subjects were asked to remain awake, lie flat at rest, breathe calmly, and keep their heads in a fixed position. First, an MRI plain scan of conventional structural images was performed to exclude subjects with brain organic lesions. The DTI was collected with a single spin echo planar imaging sequence, axial scanning, scanning a total of 45 continuous level, 12 diffusion sensitive gradient direction, the diffusion sensitive coefficient *b* = 1000, while at the same time getting an axis a scan for the best tonsure diffusion weighted imaging *b* = 0, repetition time/echo time (TR/TE) = 3600/90 ms, matrix = 128∗128, field of view (FOV) = 24∗24 cm, flip angle=90°, thickness = 0 mm. The scanning time was 4 minutes and 14 seconds.

#### 2.2.3. DTI Data Processing

The original image was converted from DICOM to NIFTI by the MRIconvert software. Based on the Matlab platform, using the PANDA to process the NIFTI data, the nonbrain tissues 3 mm away from the upper and lower, front and rear, and left and right directions of the scalp were all cut. FSL software was used for scalp stripping. The head movement correction and eddy current correction were performed on the subjects' head movements to obtain the brain template and calculate FA, based on the JHU white matter tractography atlas templates and to calculate the average 20 white matters in the region of interest (ROI) FA [11]. The raw DTI data were observed by the naked eye, and no obvious artifacts were found. The average FA in 20 ROI was extracted and placed in SPSS 23.0 for statistical analysis.

#### 2.2.4. Statistical Analysis

This study used SPSS 23.0 ANOVA was performed for age, years of education, and HAMD-24 among the three groups, and the chi-squared test was used for gender. Measurement data were expressed as mean ± SD. The test level was set at *α* = 0.05. *P* < 0.05 indicated that the difference was statistically significant. The FA extracted from PANDA was placed in SPSS 23.0 and analyzed by ANOVA, and the regions with significant differences were compared in pairs under the Least—Significant Difference (LSD). The results were considered statistically significant when *P* < 0.01. Through SPSS 23.0, a Two-sample T-test was used to compare the differences of SHAPS/FSS/RBANS between MDD and HC. Pearson correlation analysis was used to analyze the correlation between abnormal FA with statistical differences and clinical characteristics in MDD. The results in *P* < 0.05 were considered statistically significant.

## 3. Results

### 3.1. General Demographic Data

There was no statistically significant difference in gender, age, or years of education among the three groups (*P* < 0.05), but there was a statistically significant difference in the HAMD-24 score (*P* < 0.05) (see Table 1).

### 3.2. White Matter FA

#### 3.2.1. Overall White Matters FA

There were six differences in white matter in MDD, First-degree relatives, and HC, and these included LATR, RATR, LCST, FMa, RILF, and LSLF(T) (*P* < 0.01) (see Table 2).

#### 3.2.2. MDD, First-Degree Relatives, and HC Were Compared Pair-Wise

**Figure 1:**
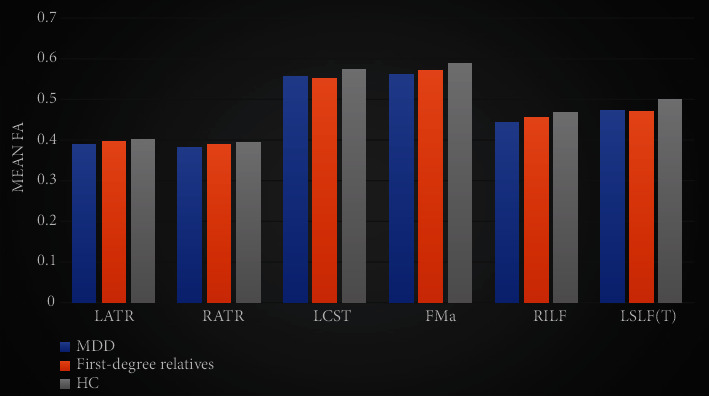
Differences in MDD, First-degree relatives and HC white matter. The bar graph represents the FA mean ± 2SD. ^∗^*P* < 0.01.

Multiple comparisons and corrections of brain regions showed that there were significant differences between MDD/HC and First-degree relatives/HC in three regions: LCST, FMa, and LSLF(T); however, there were no significant differences between MDD/First-degree relatives. The values of MDD and First-degree relatives FA were both lower than that of the HC (see Table 3 and Figure 1).

### 3.3. Correlation Analysis of White Matter Changes and Clinical Manifestations

#### 3.3.1. Differences in Clinical Manifestations between MDD and HC

MDD was significantly increased in SHAPS and FSS when compared to HC. The scores of immediate memory, visual span, speech function, attention, and delayed memory in the RBANS test of MDD were significantly lower than those of HC, and the difference was statistically significant (*P* < 0.05) (see Table 4).

#### 3.3.2. Correlation between Abnormal White Matter FA and Clinical Manifestations

**Figure 2:**
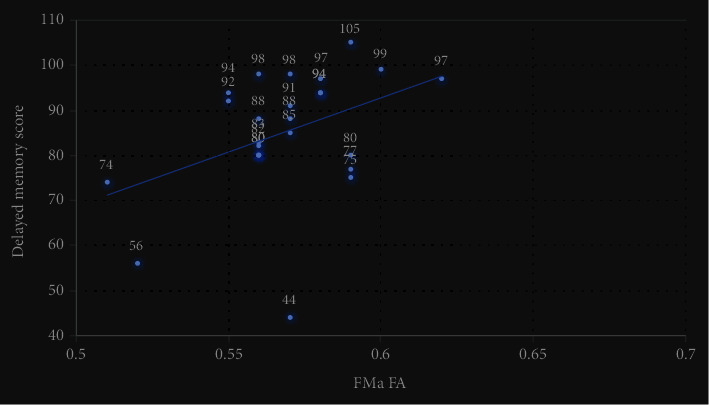
Dispersion of FMa FA and delayed memory score. FMa FA was positively correlated with a delayed memory score (*r* = 0.43, *P* = 0.031).

**Figure 3:**
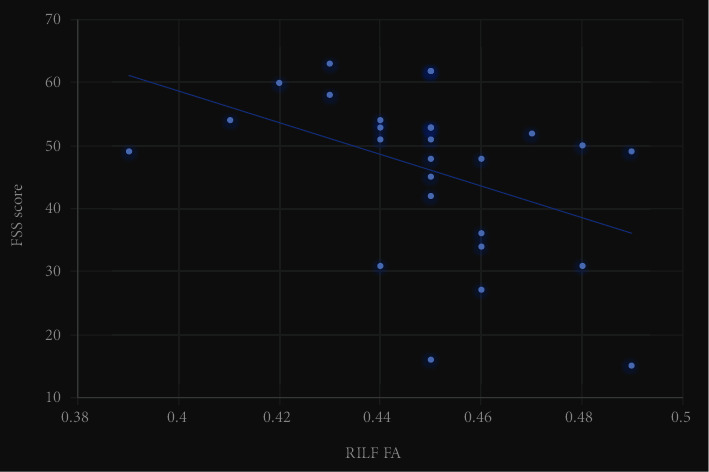
Dispersion of RILF FA and FSS. RILF FA was negatively correlated with total FSS score (*r* = −0.42, *P* = 0.028).

There was a significant positive correlation between FMa FA and delayed memory score (*r* = 0.43, *P* = 0.031), as well as a significant negative correlation between RILF FA and FSS total score (*r* = −0.42, *P* = 0.028). No significant correlation was found for the rest (see Table 5 and Figures 2 and 3).

## 4. Discussion

### 4.1. About the DTI

This study used the JHU white matter tractography atlas, based on the parameters of ROI. The JHU white matter tractography atlas divides white matter fiber tracts into 20 regions. Although this method is less sensitive than voxel-based and white matter skeleton-based, the obtained results are reliable. At the same time, rather than just carrying out correlation analysis on a certain lump of differences, our study used a ROI-based analysis method to ascertain that each brain region had clear anatomical significance [12].

### 4.2. About the Results

These results indicated that while LCST (*P* = 0.262), FMA (*P* = 0.813), and LSLF(T) (*P* = 0.878) had the same white matter characteristics in patients with MDD as in first-degree relatives, they were not found in healthy controls. Therefore, we speculate that the impairment of LCST, FMA, and LSLF(T) is a quality indicator of MDD and that the first-degree relatives of MDD patients need more state changes to develop the disease. We also found that FMa was associated with cognitive function and that RILF was associated with physical symptom.

ATR is an important component of the cortical-thalamic-cortical circuit and is mainly involved in the execution and planning of complex behaviors, which can explain why ATR changes lead to the onset of MDD [13]. Our study showed the presence of bilateral ATR damage in MDD. Previous studies showed that the FA decrease of ATR was also found in bipolar disorder (BD), indicating that ATR plays an important role in the onset of affective disorders [14], though this may be related to the different participants. The FA reduction in LCST has been widely reported in previous studies on BD, which is similar to the findings located in MDD in our study [15]. Chhetry et al. found that MDD remisses were associated with increased LCST FA [16]. Decreased FA of CST were also found in studies of patients with schizophrenia [17, 18]. There were also studies inconsistent with our results. Sacchet Matthew et al. obtained the MDD bilateral CST with a higher FA [19]. Meta-analysis showed that FMa reduction was a common feature of affective disorders [20]. Studies on MDD have also found that FA of FMa may be related to anhedonia [21], but our study did not find that, and this may be related to the heterogeneity of samples and different data processing methods. Previous studies have found ILF changes in MDD. Maurizio et al. found that ILF was significantly abnormal in MDD [22, 23]. FA abnormalities in LILF also exist in adolescent depression [24]. Reduced FA in LILF was found in all psychiatric disorders without distinguishing the disease types, and this change was related to the severity of the disease [25]. Our research also shows that first-degree relatives as high-risk groups have LILF anomaly, this may be related to the MDD recurrence. Studies have shown that the FA value of LSLF in MDD decreases, which is similar to our results [26]. FA changes in SLF may be related to the NETRIN1 signaling pathway [27]. Reduced FA in RSLF was found in individuals with a family history of BD [28], thereby suggesting a degree of heritability in RSLF changes. A previous review indicated that a lower FA value of ILF in Parkinson's disease patients leads to poor cognitive function, but our study did not show similar results [29].

There are many shortcomings in this study: the sample size should be expanded, and multiple methods were not used to verify the results. Of course, we did find brain imaging changes associated with the onset of MDD, and this provides the foundation for further research work.

## Figures and Tables

**Table 1:** General demographic data of each group.

Items	MDD (*n* = 27)	First-degree relatives (*n* = 16)	HC (*n* = 28)	*F*/*χ*^2^	*P*
Sex (female/male)	19/8	11/5	17/11	0.306	0.738
Age	28.92 ± 8.72	30.93 ± 4.15	26.78 ± 6.91	1.643	0.201
Education (year)	13.77 ± 2.48	13.56 ± 2.12	14.85 ± 2.42	2.038	0.138
HAMD-24	26.92 ± 4.15	4.56 ± 1.71	5.39 ± 1.68	475.877	<0.01

**Table 2:** White matter fiber values and differences of MDD, First-degree relatives, and HC.

Fiber	MDD FA	First-degree relatives FA	HC FA	*F*	*t*
Left anterior thalamic radiation	0.39 ± 0.01	0.40 ± 0.02	0.41 ± 0.01	6.089	0.004^∗^
Right anterior thalamic radiation	0.38 ± 0.02	0.39 ± 0.02	0.40 ± 0.01	8.754	0.000^∗^
Left corticospinal tracts	0.56 ± 0.02	0.55 ± 0.02	0.58 ± 0.02	8.436	0.001^∗^
Right corticospinal tracts	0.57 ± 0.02	0.57 ± 0.03	0.58 ± 0.02	3.358	0.041
Left cingulated	0.52 ± 0.02	0.53 ± 0.03	0.54 ± 0.03	1.468	0.238
Right cingulated	0.48 ± 0.04	0.49 ± 0.04	0.48 ± 0.03	0.399	0.673
Left hippocampus	0.40 ± 0.03	0.41 ± 0.03	0.41 ± 0.03	0.495	0.611
Right hippocampus	0.37 ± 0.02	0.43 ± 0.05	0.42 ± 0.04	0.604	0.549
Forceps major	0.57 ± 0.02	0.57 ± 0.02	0.59 ± 0.01	7.621	0.001^∗^
Forceps minor	0.44 ± 0.02	0.44 ± 0.02	0.45 ± 0.01	2.494	0.090
Left inferior frontal occipital tract	0.42 ± 0.02	0.43 ± 0.02	0.44 ± 0.02	3.363	0.040
Right inferior frontal occipital tract	0.44 ± 0.02	0.44 ± 0.02	0.45 ± 0.02	4.820	0.011
Left inferior longitudinal fasciculus	0.44 ± 0.02	0.43 ± 0.03	0.44 ± 0.02	4.327	0.017
Right inferior longitudinal fasciculus	0.44 ± 0.02	0.45 ± 0.03	0.47 ± 0.02	6.675	0.002^∗^
Left superior longitudinal fasciculus	0.37 ± 0.02	0.38 ± 0.01	0.38 ± 0.01	5.235	0.008
Right superior longitudinal fasciculus	0.39 ± 0.02	0.40 ± 0.03	0.40 ± 0.01	4.813	0.011
Left uncinate fasciculus	0.41 ± 0.02	0.40 ± 0.03	0.41 ± 0.01	0.178	0.838
Right uncinate fasciculus	0.41 ± 0.02	0.41 ± 0.02	0.42 ± 0.02	2.429	0.096
Left superior longitudinal fasciculus (temporal)	0.47 ± 0.03	0.47 ± 0.03	0.50 ± 0.04	5.996	0.004^∗^
Right superior longitudinal fasciculus (temporal)	0.52 ± 0.04	0.53 ± 0.05	0.56 ± 0.05	4.345	0.017

^∗^*P* < 0.01.

**Table 3:** Comparison between MDD, First-degree relatives, and HC.

Fiber	MDD/HC	MDD/first-degree relatives	First-degree relatives/HC
LATR	0.001^∗^	0.190	0.100
RATR	0.000^∗^	0.234	0.022
LCST	0.003^∗^	0.262	0.000^∗^
FMa	0.001^∗^	0.813	0.005^∗^
RILF	0.001^∗^	0.363	0.033
LSLF(T)	0.003^∗^	0.878	0.007^∗^

^∗^*P* < 0.01.

**Table 4:** MDD and HC clinical symptoms difference.

Items	MDD	HC	*t*
Affective symptoms	23.15 ± 6.304	4.59 ± 4.29	0.000^∗^
Physical symptom	46.185 ± 13.12	25.84 ± 5.79	0.000^∗^
Spatial span	74.20 ± 15.16	96.19 ± 14.85	0.038^∗^
Visual span	90.88 ± 20.10	103.42 ± 13.03	0.033^∗^
Speech function	88.20 ± 17.88	97.27 ± 11.41	0.000^∗^
Attentional function	99.76 ± 16.01	119.38 ± 13.29	0.004^∗^
Delayed memory	84.12 ± 16.47	95.19 ± 6.87	0.000^∗^

^∗^*P* < 0.05.

**Table 5:** Differences in clinical symptoms between MDD and HC.

Tests	Correlation between white matter and test scores					
	LATR	RATR	LCST	FMa	RILF	LSLF(T)
Affective symptoms	0.17	0.06	0.22	0.22	-0.13	0.03
Physical symptom	-0.03	-0.10	-0.12	-0.20	-0.42^∗^	0.01
Spatial span	0.13	0.05	0.06	-0.37	0.18	-0.16
Visual span	-0.22	-0.15	-0.20	0.09	0.17	-0.04
Speech function	-0.11	-0.20	-0.03	0.22	0.21	-0.17
Attentional function	-0.79	-0.34	-0.03	0.13	0.21	-0.17
Delayed memory	0.24	0.35	0.15	0.43^∗^	0.34	-0.05
Severity	0.22	0.15	0.36	0.21	0.27	-0.03

^∗^*P* < 0.05.

## Data Availability

The data used to support the findings of this study are included within the article.
